# Clinician's guide to trustworthy and responsible artificial intelligence in cardiovascular imaging

**DOI:** 10.3389/fcvm.2022.1016032

**Published:** 2022-11-08

**Authors:** Liliana Szabo, Zahra Raisi-Estabragh, Ahmed Salih, Celeste McCracken, Esmeralda Ruiz Pujadas, Polyxeni Gkontra, Mate Kiss, Pal Maurovich-Horvath, Hajnalka Vago, Bela Merkely, Aaron M. Lee, Karim Lekadir, Steffen E. Petersen

**Affiliations:** ^1^William Harvey Research Institute, NIHR Barts Biomedical Research Centre, Queen Mary University of London, London, United Kingdom; ^2^Barts Heart Centre, St Bartholomew's Hospital, Barts Health NHS Trust, London, United Kingdom; ^3^Semmelweis University Heart and Vascular Center, Budapest, Hungary; ^4^Division of Cardiovascular Medicine, Radcliffe Department of Medicine, National Institute for Health Research Oxford Biomedical Research Centre, Oxford University Hospitals NHS Foundation Trust, University of Oxford, Oxford, United Kingdom; ^5^Departament de Matemàtiques i Informàtica, Artificial Intelligence in Medicine Lab (BCN-AIM), Universitat de Barcelona, Barcelona, Spain; ^6^Siemens Healthcare Hungary, Budapest, Hungary; ^7^Department of Radiology, Medical Imaging Centre, Semmelweis University, Budapest, Hungary; ^8^Health Data Research UK, London, United Kingdom; ^9^Alan Turing Institute, London, United Kingdom

**Keywords:** artificial intelligence, cardiovascular imaging, machine learning (ML), trustworthiness, AI risk

## Abstract

A growing number of artificial intelligence (AI)-based systems are being proposed and developed in cardiology, driven by the increasing need to deal with the vast amount of clinical and imaging data with the ultimate aim of advancing patient care, diagnosis and prognostication. However, there is a critical gap between the development and clinical deployment of AI tools. A key consideration for implementing AI tools into real-life clinical practice is their “trustworthiness” by end-users. Namely, we must ensure that AI systems can be trusted and adopted by all parties involved, including clinicians and patients. Here we provide a summary of the concepts involved in developing a “trustworthy AI system.” We describe the main risks of AI applications and potential mitigation techniques for the wider application of these promising techniques in the context of cardiovascular imaging. Finally, we show why trustworthy AI concepts are important governing forces of AI development.

## Introduction

In recent years, several artificial intelligence (AI) based systems have been developed in cardiology. This trend is driven by the increasing need to deal with the vast amount of clinical and imaging data produced in the field and with the ultimate aim to advance patient care, diagnosis and prognostication (1, 2). It is not a question anymore whether AI will transform healthcare but rather how it will do so (3). Transformative measures have already impacted many areas of cardiovascular medicine, from smart devices promising to diagnose arrhythmias based on single-lead ECG (4) to automatic image segmentation tools shortening manual image analysis (5, 6). However, there is a critical gap between the development and deployment of AI tools. To date only 24 AI-driven cardiovascular imaging products have received FDA approval (7), suggesting there remain critical challenges in building and implementing these models into everyday practice.

It is easy to scare away busy clinicians with endless legal documentation and specialized terms from philosophy, law and data science. On the other hand, expecting the data science community to be up to date with their field, understand complex medical concepts and consider the ethical ramifications of AI is the recipe for serious unintended consequences (8). Indeed, the discussion around the ethical issues of AI should be inclusive of all participants, from funding agencies to the patients.

The promise of AI revolutionizing cardiovascular imaging could not be delivered without achieving the trust of the end-users and patients. Currently, there are several ethical frameworks for AI applications. One of the most universal guideline was proposed by the European Commission in 2019 (9). This document provides a detailed technical summary and general guidance for dealing with the ethical questions of AI. However, it was written by senior data scientists, consequently does not focus on issues of healthcare applications (10). Indeed, to date, little is accessible to healthcare professionals without an in-depth understanding of the technical terms of the ethical questions embedded in AI applications. Notably, the document written by the European and North American Societies in Radiology detailing potential AI ethics issues can work as a primer for other societies in medicine (11). More recently, the first comprehensive guideline for assessing the trustworthiness of AI-based systems in medical imaging was developed, named FUTURE-AI (12). This technical framework promises to transform AI development in medical imaging and will help create an environment for safe clinical implementation of novel methods (https://future-ai.eu/).

In this narrative review, we aim to summarize the main risks of AI application and potential mitigation techniques in plain language. We provide an overview of ongoing efforts to improve the “trustworthiness” of AI in cardiovascular imaging. Finally, we aim to provide key questions to help initiate dialogue within research groups.

## The basic concepts of AI

Several dedicated publications describe AI's definitions and main applications within cardiology in great detail (13–16). Here we restrict ourselves to those basic concepts essential for further discussion of AI trustworthiness.

*AI* is an umbrella term within data science, incorporating a wealth of models, use cases and aiding methodologies to mimic human thought processes and learning patterns (8). Within AI, the most commonly used models are *machine learning (ML)*-based in medical research; therefore, several important source documents handle AI and ML almost synonymously (1). An overly simplified definition of ML is computer algorithms that “learn” from data. ML methods use pre-processed (e.g., anthropometric data derived from patients) and raw data (e.g., raw imaging files). *Deep learning (DL)* is a subset of ML that deals with algorithms inspired by the structure and function of the human brain. DL algorithms use neural networks to transform the raw data into an abstract level, refine accuracy and adjust when encountering new data (17).

We can differentiate between supervised and unsupervised learning based on the type of data fed into an AI algorithm. In *supervised learning*, humans curate and label data before training, and the model is optimized for accuracy with known inputs and outputs. The following models are used for: classification (putting data into categories) and regression (predicting continuous variables within the concept of ML). On the other hand, *unsupervised learning* deals mainly with unlabelled data, with the ultimate goal of identifying novel patterns in a dataset such as clustering (14).

A critically important step in ML model development is a large and consistently labeled data set—the diverse quality of data and the inconsistent labeling could reduce the accuracy of AI model. Another important step is data splitting: datasets are generally split into training, validation and test sets. Training and validation sets used to train and fit the model, more specifically the validation provides an estimation of the model fit for model selection or tuning of parameters, whilst the test set is reserved to evaluate the final model (18). Given the degradation in performance reported for deep-learning algorithms for medical imaging, it is of paramount importance that the test set consists of independent cohorts to allow for *external validation*, a key requirement for ensuring the trustworthiness of AI systems (19, 20). Moreover, the external validation should be performed by independent parties to ensure objectiveness. Please note, that validation in the original dataset is not synonymous with external validation, which is performed on a separate dataset.

## The concept of trust and trustworthiness of AI in medicine

It is easy to get lost in a philosophical discussion about how to define trust or if it is even possible outside the human realm (21, 22). From a practical standpoint, these questions are confusing rather than helpful. For decades AI was part of the scientific discussion, existing in research environments, and science fictions. Therefore, the question of whether to trust AI tools in healthcare was merely a discussion for scholars in the ethical and data science fields. However, with novel tools emerging daily, we are forced to reconsider the potential ramifications embedded in AI.

The questions we face today are highly practical and directly affect the field's development. Can we trust the CMR segmentation provided by the AI tool? Are we confident that the new artifact-removing algorithm does not mask any important clinical clue? Should we rely on the novel diagnosis support toolkit? What does it mean to trust the judgement of an automatic tool? How do we communicate the uncertainties embedded in a novel predictions score? Are we holding AI to a higher standard than clinical judgement based on intuition and experience?

As an example, left ventricular ejection fraction (LVEF) measured using echocardiography is a long-standing “trusted” parameter in cardiovascular medicine. Because with years of development, validation, and experience, we learned to comprehend the signals that link it to disease and outcome, and communicate the findings to the patients so that they trust their practitioners to understand echocardiography (23, 24). Although the information it provides is far from complete and prone to errors, the usefulness of knowing the EF of a patient in a clinical situation is beyond question; even when clinicans use eyeballing (25). On the other hand an AI application based on the idea that the human eye and brain can learn with experience how to estimate EF without measuring ventricular volumes and making calculations is more controversial, as this approach does not allow the revision of the ventricular contours in case of seemingly disparate results (26).

Wynants et al. reviewed multivariable COVID-19-related prediction models at the beginning of the pandemic. They found that the 232 models identified in the study all reported moderate to excellent predictive performance, but all were appraised to have a high or uncertain risk of bias owing to a combination of poor reporting and poor methodological conduct for participant selection, predictor description, and statistical methods used (27). The most sobering conclusion was that none of the proposed models proved to be of much help in clinical practice. The same conclusion was drawn from the investigation by the Alan Turing Institute (28) and others (29).

## Main AI applications within cardiovascular imaging

Within cardiovascular imaging, the main areas of AI application are: (1) image acquisition and reconstruction—which helps to reduce the scan time, (2) improving the imaging workflow and efficiency of time-expensive tasks such as segmentation, (3) improving the diagnosis-making process, (4) evaluation of disease progression and prognosis, (5) assessment of treatment effectiveness, and (6) generation of new knowledge. Examples illustrating key areas of AI applications from non-invasive cardiovascular imaging is summarized in Table 1, further examples in can be found in dedicated publications (15, 16, 18, 49, 50).

**Table 1:** Examples of AI applications from non-invasive cardiovascular imaging.

**AI application**	**Purpose**	**Modality**	**References**
Image acquisition and reconstruction	Improving image quality, decreasing image artifacts	CCTA	Wolterink et al. (30)
		CMR	Oksuz et al. (31)
	Lowering radiation dose	CT	Benz et al. (32)
	Increasing imaging speed	CMR	Caballero et al. (33)
	Improving non-expert usage (e.g., view classification, automated planning)	Echocardiography	Zhang et al. (34)
		CMR	Edalati et al. (35)
Improving the imaging workflow and efficiency of time-expensive tasks	Automatization of previously manual tasks for increased speed, effectiveness, and potentially improved standardization (e.g., image segmentation)	Echocardiography	Leclerc et al. (36)
		CCTA	Huang et al. (37)
		CMR	Bai et al. (5)
Diagnosis making	Supporting early diagnosis and timely treatement initiation or prevention	Echocardiography	Sengupta et al. (38)
		CCTA	de Vos et al. (39)
		CMR	Zhang et al. (40)
Disease prognostication	Improving the discrimination of high risk imaging features	Echocardiography	Samad et al. (41)
		CCTA	Patel et al. (42)
		CMR	Cheng et al. (43)
Assessment of treatment effectiveness	Monitoring response to medication, device therapy etc.	Echocardiography	Tokodi et al. (44)
		CCTA	Queirós et al. (45)
Generation of new knowledge	Discovering new patterns, cardiovascular phenotypes or disease presentations (key role for unsupervised learning methods)	Echocardiography	Casaclang-Verzosa et al. (46)
		CCTA	Hoshino et al. (47)
		CMR	Zheng et al. (48)

CCTA, coronary computed tomography angiography; CMR, cardiovascular magnetic resonance.

There has been a steep increase in publications using ML in cardiovascular imaging in the past 5 years. This trend was driven by the increasing availability of high computational power, large datasets (16), and the discovery of the computational effectiveness of convolutional neural network architecture (AlexNet) (51).

It has been envisioned that AI tools will take over or at least substitute the work of radiologists and cardiovascular imagers to a great extent and consequently necessitate fewer human resources creating cheaper and more accurate care in the future (52). Roughly a decade into the area of accessible AI innovation, we can see that changes are less rapid, and the results are beneath our expectations (53). No segmentation is used unchecked, no diagnosis is made without human supervision and approval, and the need for well-trained imagers has increased (54). Notably, only a small proportion of the proposed methods, models and tools gain approval from the appropriate authorities (FDA or European Medicines Agency), and reach the clinical application stage. Should we then just conclude that AI is pointless and we must not use it? On the contrary, these experiences and setbacks should motivate the research into more robust AI models and rigorous validation standards. Only by learning from the critical issues raised by researchers and end-users can we move forward in the field of AI.

## Unintended consequences of AI applications in cardiovascular imaging and mitigation strategies

To understand why AI applications are not approved and used to the rate it was predicted during the height of the ML hype in 2016, we have to look into the potential limitations of these tools. Here we provide an introduction to the main risks of AI applications within cardiovascular imaging: (1) lack of robustness and reliability causing patient harm, (2) issues of AI usability and the misuse of tools, (3) bias and lack of fairness within the AI application which can perpetuate existing inequities, (4) privacy and security issues, (5) lack of transparency, (6) gaps in explainability, (7) gaps in accountability, and (8) obstacles in implementation (Table 2). In each section, we describe the main attributes of each risk, provide relevant examples within cardiovascular imaging and illustrate potential mitigation strategies.

**Table 2:** Questions to promote discussion of AI trustworthiness between clinicians and technical experts.

**Robustness and reliability**
•Did you perform any pre-processing that can potentially affect the reliability of your models? •Did you use homogenous/ single center data OR heterogenous/multicenter data?
Are there any checkpoints for quality control in your pipeline?
**Usability**
•Do you have an understanding of the end-users needs in terms of the tool's interface? •Does the implementation of your tool viable within the clinical workflow?
**Bias and fairness**
•What fairness means for your application? •Are there any potential hidden sources of bias? •Does the algorithm exhibit discrimination toward any group? Is it harmful or beneficial for the use case? •Did you document and report these potential biases?
**Security and safety**
•Did you document potential risks of your AI tool? How do you communicate these? •Does the implementation of your AI can potentially harm patients, worsen outcome or create security breach? If not, how do you know?
**Transparency**
•Did you document the characteristics of your dataset? •Did you follow any relevant reporting guideline or checklist?
**Explainability**
•Do you know what level of explainability your end-users require? •Can you explain, how your model reaches a certain decision? •Did you explore complementary explainability methods?
**Accountability**
•What are the relevant regulations in terms of liability in your use case? •Who is responsible for errors occurring during the clinical application of the AI tool? •Who is monitoring the application and how frequently?

Summary of potential questions to support the discussion of AI trustworthiness between clinicians and technical experts adapted from Ammanath (8) and Lekadir et al. (55).

### Robustness and reliability

AI robustness is defined as the ability of a system to maintain its performance under changing conditions (56). The promise of a robust AI tool is that it can consistently deliver accurate outputs, even when it encounters unexpected or subquality data. When a model's functionality and accuracy change easily, it is considered “brittle” (8).

Medical imaging encapsulates a wealth of potential sources for AI brittleness (12):

(1) Heterogeneity within imaging types of equipment and vendors.
(2) Image acquisition heterogeneity within imaging centers and operators.
(3) Patient-related heterogeneity (including clinical status and anthropometric peculiarities).
(4) Data labeling and segmentation heterogeneity between annotators.

As mentioned above, ML algorithms play an increasingly important role in the image acquisition of all cardiovascular imaging modalities. However, these applications are not without certain limitations. For example, Antun et al. (57) highlighted possible sources of instability of deep learning algorithms at CMR reconstructions. The instabilities usually occur in several forms e.g., undetectable perturbations may result in artifacts in the reconstruction, or a small structure like tumors may not be captured in the reconstruction phase.

The potential brittleness of AI tools is also very well-illustrated by the recent developments in CMR image segmentation (58). Critically, DL-based segmentation tools are often trained and tested on images from single clinical centers, using one vendor with a well-defined protocol resulting in homogenous datasets (59, 60). Furthermore, CMR protocols across prominent multi-center cohort studies are also standardized, prohibiting wider generalizability (5, 61, 62). A notable effort to develop segmentation tools on more heterogeneous datasets to promote robust AI tool development is the Multi-Center, Multi-Vendor and Multi-Disease Cardiac Segmentation (M&Ms) Challenge (63). Investigators of the euCanSHare international project established an open-access CMR dataset (six centers, four vendors, and more than nine phenotype groups) to enable generalizable DL models in cardiac image segmentation. The Society of Cardiovascular Magnetic resonance Imaging (SCMR) registry (64) and Cardiac Atlas project (65) are also aimed at providing diverse databases for similar research ambitions. These efforts are still ongoing, and Campello et al. (63) noted that further research is necessary to improve generalizability toward different scanners or protocols.

Automated coronary computed tomography angiography (CCTA) segmentation faced similar challenges in the past decade. Although the accuracy of the CCTA plaque segmentation tools has been validated against the gold standard invasive methods, the interplatform reproducibility remains disputed (66, 67). Indeed, the time-consuming and labor-intensive nature of quantitative plaque assessment is still responsible for the frequent visual evaluation of coronary artery disease in clinical practice, despite some emerging solutions (68).

Apart from well-curated diverse datasets for benchmarking of segmentation algorithms and the development of novel segmentation tools, the reliability of the output is also a critical to the clinical implementation of these tools. Recently, automated quality control tools have been suggested in high-volume datasets where manual expert inspection is not achievable. Automated quality control tools utilizing different methods, such as Dice similarity coefficient, reverse classification accuracy (RCA) framework, and quality control-driven (QCD) framework, have been implemented within ventricular (69), T1 mapping (70), aortic (71), coronary, and pericardial fat segmentation (72).

AI robustness largely relies on the adaptability of a given model to changing circumstances. A segmentation tool might perform well in a given dataset of healthy hearts, but it might not directly translate into a heterogeneous dataset. The following concepts help promote robustness and reliability in medical imaging applications of AI:

(1) Heterogeneous training data (multi-center, multi-vendor, multiple diseases).
(2) Checking intra- and interobserver variability and whether automated AI tool difference lies within the observer variability.
(3) Applying well-established annotation with powerful annotation software.
(4) Image quality control (to identify artifacts within the data, applying algorithms which help to reduce artifacts).
(5) Applying image harmonization techniques (including the use of phantoms and dedicated harmonization tools such as histogram normalization).
(6) Applying feature harmonization techniques (using test-retest studies and feature selection methods to select stable, robust features for the models).
(7) Data augmentation.
(8) Uncertainty estimation [there is a variety of uncertainty quantification methods, including prediction intervals, Monte Carlo dropout, and ensembling; they are designed to pick up the distance of the new observation to observations the algorithm has already seen Kompa et al. (73)].

Potential issues that can arise during the assessment of robustness and clinical usability is well-illustrated by the adaptation of radiomics in cardiovascular imaging (74). Radiomics enable the extraction of voxel-level information from digital images, promising the quantitative description of tissue shape and texture. The utility of CT radiomics has been demonstrated in identifying vulnerable coronary atherosclerotic plaques (75–77) and linking pericoronary adipose tissue patterns to local inflammation (78, 79). CMR radiomics has also been shown to improve the discrimination of cardiomyopathies (80–82) and improve risk prediction among ST-elevation myocardial infarction patients (83, 84). Despite these advances, the clinical implementation of radiomics is in its infancy. The general critique of the technique lies in the poor repeatability of radiomics features. To improve radiomics usability in CMR, Raisi-Estabragh et al. (85) conducted a multi-center and multi-vendor test-retest study to evaluate the repeatability and reproducibility of CMR radiomics features using cine imaging. The authors reported variable levels of repeatability of the features, which are likely to be clinically relevant. To reduce the radiomics variability introduced by the acquisition center Campello et al. (86) evaluated several image- and feature-based normalization techniques. The authors demonstrated that ComBat, a feature-based harmonization technique, can remove center information, but this does not translate to better algorithmic generalization for classification. The best performing approach in this respect was piecewise linear histogram matching normalization.

### Usability

Usability is defined as the extent to which an AI application can be utilized to achieve specific goals by specified users with effectiveness, efficiency and satisfaction (87). As the interaction between healthcare professionals and technology is increasingly important, more and more research effort is aimed at testing clinical usability. However, AI tools are barely tested regarding how they interact with clinicians, and most applications are still in “proof-of-concept” status (88). Key issues of usability include lack of a human-centered approach for the development of the AI technologies, e.g., lack of involvement of the end-user for the definition of the clinical requirements and of multi-stakeholder engagement throughout the production lifecycle.

### Bias and fairness

In AI, defining bias and fairness is challenging due to the ever-changing applications we put AI to ISO/IEC TR 24027:2021 (89). Within the healthcare domain, fairness means that AI algorithms should be impartial and maintain the same performance when applied to similarly situated individuals (individual fairness) or different groups of individuals, including under-represented groups (group fairness) (12).

Until now, little data is available regarding the bias and fairness of algorithms in cardiovascular imaging, even though the phenomenon is well-known. As Rajkomar et al. summarized: any type of bias depicted within the dataset is learned and adapted into model performance (90). Overrepresentation of a certain group leads to data collection bias (18), as exemplified by Larrazabal et al. (91). They demonstrated in a large-scale analysis of chest X-ray images that gender imbalance in the training dataset led to incorrect classification of important conditions such as atelectasis, cardiomegaly or effusion. Puyol-Antón et al. performed the first analysis of DL fairness in cardiovascular segmentation using the UKB dataset (92). They found that the segmentation algorithm trained on a dataset balanced regarding participant sex but imbalanced concerning ethnicity resulted in less reliable outcomes for minority groups. It is easy to see how data biases might lead to a less inclusive distribution of resources. Lack of fairness might not only lead to loss of opportunities and worse health outcomes among minority groups but may also reduce public trust in AI applications.

Lekadir et al. identified the main guiding principles for fairness in medical imaging AI (12). Actions to promote AI fairness are not one step but should be implemented throughout the AI lifecycle. Here we summarize the main recommendations from a clinical perspective:

(1) Multi-disciplinarity, which stands for the inclusion of all important stakeholders (AI developers, imaging specialists, patients, and social scientists) in the AI design and implementation.
(2) Context-specific definition of fairness with regards to potential hidden biases in the dataset and data annotators.
(3) Standardization of key variables (e.g., sex, and ethnicity should be collected in a standardized way, because these descriptors of the groups can help test, and verify AI fairness).
(4) The data should be probed for (im)balances, particularly participant age, sex, ethnicity, and social background.

Once we know the potential biases, we have several options to deal with them. There are tools to promote AI fairness on a data collection and curation level, as well as in the model training and testing process.

(1) Data collection process in itself should be transparent and well-documented.
(2) Collecting multi-center data.
(3) Application of specialized statistical methods to evaluate fairness (e.g., true positive rate disparity, statistical parity group fairness, equalized odds, predictive/equality) (93, 94).
(4) Application of specialized statistical methods to mitigate bias (e.g., re-sampling, data augmentation, development of stratified models by sex, or ethnicity).

Exploratory data analysis is also a great tool to probe the dataset for hidden biases (8) and should not be a solitary task for the data scientist. Researchers with a medical background are more adept at picking up chance associations and odd correlations within the dataset. Among other things, data scientists can produce synthetic data to compensate for missing values to create a more balanced dataset. At the same time, we must always stay vigilant to the potential biological meaning of missing data before deciding to make up for it. Therein lies another strong argument for inclusive AI research.

### Privacy and security

Any potential breach in healthcare AI systems can seriously undermine the trust of end-users. Therefore, developers should cooperate with cybersecurity experts to protect personal information against bad actors before clinical implementation. In some critical areas, such as data protection, there are firmly outlined rules in place: e.g., EU General Data Protection Regulation (GDPR) or the California Privacy Rights Act (CPRA). However, these regulations can never keep up with the speed of innovation.

Key issues surfacing with the use of clinical data for AI development (95):

(1) Sensitive data being shared without informed consent.
(2) Inappropriate informed consent forms (e.g., information within the consent form is detailed beyond the processing capability of the patient/user, no dedicated time allocated for consent review, and opaque use cases permitting patients from understanding how their data might be used).
(3) Data re-purposing without the patient's knowledge and consent.
(4) Personal data being exposed.
(5) Attacks on AI applications (e.g., data poisoning, adversarial attacks).

For example, the South Denver Cardiology Associates recently confirmed a data breach affecting 287,000 patients. The stolen dataset contained dates of birth, Social Security numbers, driver's license numbers, patient account numbers, health insurance information, and clinical information (96). This leakage might result in identity theft, insurance fraud or other inappropriate use of sensitive data. Moreover, in the field of medical imaging, particular attention is necessary toward dealing with potential adversarial attacks (97), including “one-pixel” attacks (98). These attacks involve slight changes to the input images intending to fool the AI and produce a false result. In other cases details of large scale data sharing agreements remain gray for the public (99), which might lead to data privacy controversies in the future.

Fortunately, several steps can be taken to mitigate these risks on an individual and institutional level:

(1) Increasing the awareness of privacy and security risks, informed consent and cybersecurity through (self)education.
(2) Transparent regulations of data privacy, data re-purposing.
(3) De-centralized, federated learning approaches such as federated learning. Federated learning is an ML setting where many de-centralized clients collaboratively train a model under the arrangement of a central server, keeping the data in several individual locations (100). Despite this, some researchers might be hesitant to use federated learning, because of the potential disclosure of the model. However, the data is never exposed to third parties, not even to the data scientist.
(4) Ongoing cybersecurity research into novel, more secure algorithms.

### Transparency and traceability

Transparency in AI is a broad term; it refers to the information about the dataset, processes, uses, and outputs that is a prerequisite for accountability. AI transparency within medicine aims to provide all stakeholders with enough information to join in the discussion in a meaningful way. Two universal requirements guide and promote AI transparency:

(1) Data transparency includes transparent methods and guidelines for data collection, utilization, storage, sharing, and documentation.
(2) Model transparency means we have enough knowledge/information about a model's internal properties to apprehend its output meaningfully.

The goal of traceability is to document the entire development process and to monitor the behaviour and functioning of an AI model or system over time. This approach allows tracking any drift from the original training settings. As clinical practice constantly evolves, images provide greater granularity or novel guidelines emerge; keeping track of the model performance and adapting it to the new circumstances is critical (101). Two main concepts driving a decrease in model performance over time are “concept drift” and “data drift”. Concept drift means that some underlying characteristics of variables change (for example, a novel type of cardiomyopathy is distinguished, creating a new class for a classification algorithm), which decreases the accuracy of the model. Data or dataset drift refers to the change in the data, meaning that a difference in the scanning device or image acquisition may directly affect the prediction model deployed (12).

Standardized dataset documentation methods can facilitate ML results' transparency, accountability and repeatability. Recently, Gebru et al. posed a list of questions on how and why data was collected, what is the composition of the data, and how it was curated and labeled in their document entitled “Datasheets for Datasets” (102). Sendak et al. proposed the use of “Model facts cards” for each ML model to ensure that clinicians have a thorough understanding of “how, when, how not, and when not to” incorporate the output into their decisions (103).

### Explainability

In terms of an AI system, explainability means that it is possible to comprehend how the output was reached. The greater the explainability of a model, the better we can understand the internal mechanisms of a decision-making tool. However, as Arbelaez Ossa et al. (104) point out, the key issue in AI explainability is the lack of consensus among data scientists, regulators, and healthcare professionals regarding the definition and requirements.

Notably, explainability is not necessary for all ML models. Simple rule-based models applying linear regression or decision trees are inherently explainable. If we can calculate how a given parameter is weighted within the model, it is unnecessary to push the limits of explainability further.

From a strictly clinical end-user perspective, it is also not necessary to understand all steps involved in a complex DL network if the output is readily accessible and visually verifiable by a physician, such as segmentation. On the other hand, if the algorithm promises to deliver a clinical diagnosis or prognostic information based on imaging data or a combination of imaging features, the clinical application needs to reach high levels of intelligibility. A clinician who does not understand how the algorithm reached its conclusion will likely to rely on their own expertise rather than an opaque output.

To deal with the “black box” nature of particularly DL methods, several *post-hoc* explainability algorithms were defined to create more interpretable models. The so-called saliency maps or heat maps are the most widely adopted explainability tools in medical imaging. These color-coded maps show the contribution of each image region to a given model prediction (105, 106). Several distinct approaches can be utilized, such as Gradient-weighted Class Activation Mapping (Grad-CAM) (105) or Dense Captioning (DenseCap) (107), to capture the most crucial image areas. Saliency maps have long been applied in image analysis to understand better the key areas supporting the model's decision. As an example, Candemir et al. (108) trained a 3-dimensional convolutional neural network (CNN) to differentiate between coronary arteries with and without atherosclerosis and has shown the essential features learned by the system on color-coded maps. Saliency maps can also suggest if an algorithm picks up temporal data: Howard et al. (109) applied time distributed CNN model with saliency maps for disease classification based on echocardiography images. The author found that these new architectures more than halve the error rate of traditional CNNs, possibly because of the networks' ability to track the movement of specific structures such as heart valves throughout the cardiac cycle.

Local Interpretable Model-Agnostic Explanations (LIME) is used to explain the model locally for one single subject (110). LIME evaluates a given variable's contribution to the whole of the predictive model. SHapely Additive exPlanations (SHAP) is a model agnostic explainability model (can be used to interpret any model) (111). SHAP is based on game theory and can reveal each predictor's effect on the outcome. It calculates a score for each feature in the model, showing the feature's size and direction effects on the outcome. Al'Aref et al. (112) applied boosted ensemble algorithm (XGBoost) in the participants of the CONFIRM registry and showed that incorporating clinical features (e.g., age, sex, cardiovascular risk factors, laboratory values, and symptoms) in addition to coronary artery calcium score can accurately estimate the pretest likelihood of obstructive coronary artery disease on CCTA. They could supply the 20 most crucial features supporting the model's prediction using the SHAP method. Similarly, Fahmy et al. (113) applied the SHAP to support the interpretation of their model looking into the association between CMR metrics and adverse outcomes (cardiovascular hospitalization and all-cause death) in patients with dilated cardiomyopathy. Many other explainability techniques are also available, and new tools are likely to become more sophisticated and model-specific.

Although these models can improve model interpretation, their understanding requires additional efforts from the physicians. We are yet to see if their outputs can become as acceptable to the community and if they can overcome current limitations. Critiques of current explainability models warn that the performance of the explanations are not routinely quantified, and we can rarely elucidate if a given decision was sensible or not (114). Moreover, they might reduce the complexity of a model to a level that is not representative and promote a false sense of security among users. Ghassemi et al. note that with the currently available methods, our best hope is to go through rigorous internal and external validation and use explainability models for troubleshooting and system audits (114).

### Accountability and liability

Accountability refers to the state of being responsible. However, in the context of AI, where algorithms are based on both ML and human ingenuity, the mistakes or errors of the application come from humans developing or using machines (95, 115, 116). On the other hand, it is not clearly defined and regulated yet, with whom the responsibility of AI-powered medical tools lies. Does liability fall on developers, chief executive officers of the developing company, leaders of the healthcare institution buying and authorizing clinical utilization or the doctors using them? Notably, sometimes it is also hard to pinpoint why the AI-related medical error happened (95); therefore, responsibility issues can lead to daunting detective work, steering away the attention from the actual patient care. The main proposed tools to mitigate accountability issues within AI are: (1) the roles and responsibilities of developers and users should be defined, (2) a regulatory framework for accountability should be in place, and (3) dedicated regulatory agencies should be established and monitor AI use.

### Clinical implementation

Even if an AI tool complies with all of the criteria mentioned earlier, integrating a new tool into clinical practice hides several expected and unexpected difficulties. The main obstacles to clinical implementation stem from three primary sources: (1) the differences among institutions regarding equipment, staffing, location, financial possibilities, and inner structures of each healthcare institution, (2) change in physician-patient relationship, and (3) difficulties of clinical and technical integration into existing workflows (95, 117).

Medical data, in general, is very noisy and requires human oversight before integration. Cardiovascular imaging data is slightly more structured than clinical records but still lacks interoperability to a great extent (118). Several initiatives already aim at increasing interoperability among healthcare providers [e.g., European Commission (119), Health Data Research UK (120)]. However, it seems fairly evident that medical AI tools will have to adapt to a certain level of data heterogeneity. The physician-patient relationship has been transformed by technical advances and the maturity of social sciences, but it is yet uncertain how AI tools will impact this relationship. Some argue that it will help by easing clinician workload and providing more personalized data for shared decision-making, while others question doctors' role once critical tasks are delegated to sophisticated algorithms (121). Clinical guidelines will need to be updated to consider the potential role of AI tools between healthcare workers and patients (95, 122). Moreover, these guidelines will also need to be updated to integrate novel tools into the clinical workflow without severe disruption of care (123).

## Actionable steps

**Figure 1:**
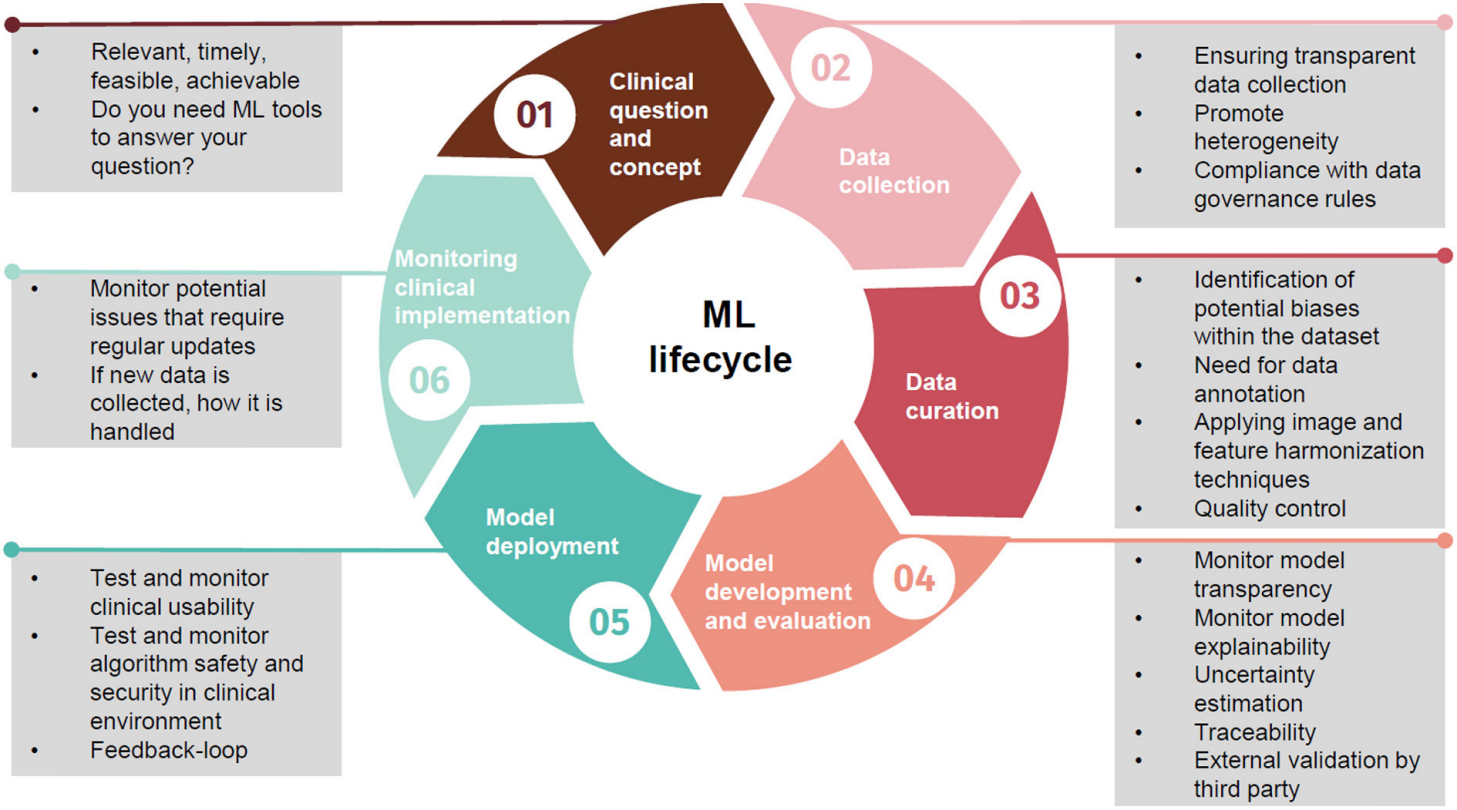
Principles of trustworthy AI within the machine learning lifecycle.

Trustworthy AI is not an obscure concept reserved for technical specialists and scholars of ethical reals (124), but rather a practical set of steps and questions, which, when implemented, can provide us with reliable tools for a new era in healthcare. In an effort to improve the overall quality of the AI prediction models, van Smeden et al. presented 12 critical questions for cardiovascular health professionals to ask (125). Moreover, the use of the Proposed Requirements for Cardiovascular Imaging-Related Machine Learning Evaluation (PRIME) checklist has been suggested by Sengupta et al. (126), a framework that contains a comprehensive list of crucial responsibilities that need to be completed when developing ML models. Here we report key questions to promote discussion of AI trustworthiness between clinicians and technical experts (Table 2), moreover we summarize how these principles fit into the ML lifecycle (Figure 1).

We have to acknowledge that in some instances medical research and consequently medical AI research is plainly inaccurate, but we can rectify these mistakes over time. AI competitions provide an excellent platform for robust validation or rebuttal of results. As an example, a recent competition to predict O(6)-Methylguanine-DNA-methyltransferase (MGMT) promoter methylation from brain magnetic resonance imaging (MRI) scans (127). Overall, 1,555 teams of many thousands of researchers took a large dataset of MRI scans and the results clearly demonstrate that this task is not possible with current approaches, even tough several group claimed to have achieved an ROC scores of up to 0.85 previously (128–130). This suggest that well designed competitions provide and excellent opportunity to improve the quality of AI research.

In order to promote the safe adoption of AI-powered tools in cardiovascular imaging, practicing doctors and future medical professionals need to be properly trained in the technical aspects, potential risks and limitations of the technology (131). McCoy et al. (132) and Grunhut et al. (133) proposed crucial points to improve AI literacy in medical education programs. Furthermore, the involvement and education of the general public are also essential for the broader adoption of these emerging tools.

Embracing the human-in-the-loop principle may offer further benefits where both imagers and ML algorithms fall short (134). It means that we can benefit from the advantages of AI models (i.e., automated segmentation or diagnosis) and having a human at various stages or checkpoints to correct potential errors or use critical thinking where algorithms are not confident in their results. The human can validate or correct the results where the algorithm delivers lower confidence outputs, creating a combined and better decision.

In essence, it does not matter if we call it trustworthy AI, reliable AI or responsible AI—the driving idea is to create an inclusive, collaborative effort in healthcare between all stakeholders. Our task is to consider the possible impact and test our AI tool and all elements of the AI development by posing the right questions relevant to our desired aims.

## Author contributions

ZR-E, KL, SEP, and LS contributed to the conceptualization of this review. LS wrote the original draft and produced the table and figure. ZR-E, SEP, PM-H, HV, BM, and AML reviewed the manuscript from a clinical perspective. AS, CM, ER, PG, MK, AML, KL, and SEP reviewed the manuscript from a technical perspective. All authors contributed to the article and approved the submitted version.

## Funding

LS received funding from the European Association of Cardiovascular Imaging (EACVI Research Grant App000076437). ZR-E recognizes the National Institute for Health Research (NIHR) Integrated Academic Training Programme which supports her Academic Clinical Lectureship post and was also supported by British Heart Foundation Clinical Research Training Fellowship Grant No. FS/17/81/33318. AS was supported by British Heart Foundation project grant (PG/21/10619). CM was supported by the Oxford NIHR Biomedical Research Center. SEP acknowledges support from the SmartHeart EPSRC programme grant (www.nihr.ac.uk; EP/P001009/1) and also from the CAP-AI programme. CAP-AI is led by Capital Enterprise in partnership with Barts Health NHS Trust and Digital Catapult and was funded by the European Regional Development Fund and Barts Charity. SEP, PG, and KL have received funding from the European Union's Horizon 2020 research and innovation programme under grant agreement no. 825903 (euCanSHare project). HV, LS, and BM acknowledge funding from the Technology of Hungary from the National Research, Development, and Innovation Fund, financed under the TKP2021-NKTA funding scheme and Project No. RRF-2.3.1-21-2022-00004 (MILAB) which has been implemented with the support provided by the European Union. AML acknowledges support from the ‘SmartHeart' EPSRC programme grant (www.nihr.ac.uk; EP/P001009/1) and from the UKRI London Medical Imaging and Artificial Intelligence Centre for Value Based Healthcare. This article is supported by the London Medical Imaging and Artificial Intelligence Centre for Value Based Healthcare (AI4VBH), which is funded from the Data to Early Diagnosis and Precision Medicine strand of the government's Industrial Strategy Challenge Fund, managed and delivered by Innovate UK on behalf of UK Research and Innovation (UKRI). ER was partly funded from the programme under grant agreement no. 825903 (euCanSHare project) and grant agreement no. 965345 (HealthyCloud project). The funders provided support in the form of salaries for authors as detailed above but did not have any additional role in the study design, data collection and analysis, decision to publish, or preparation of the manuscript.

## Author disclaimer

Views expressed are those of the authors and not necessarily those of the AI4VBH Consortium members, the NHS, Innovate UK, or UKRI.

## Conflict of interest

Author SEP provides consultancy to Cardiovascular Imaging Inc, Calgary, Alberta, Canada. Author MK was employed by Siemens Healthcare Hungary.

The remaining authors declare that the research was conducted in the absence of any commercial or financial relationships that could be construed as a potential conflict of interest.

## Publisher's note

All claims expressed in this article are solely those of the authors and do not necessarily represent those of their affiliated organizations, or those of the publisher, the editors and the reviewers. Any product that may be evaluated in this article, or claim that may be made by its manufacturer, is not guaranteed or endorsed by the publisher.
